# The Ins and Outs of B Cells in Multiple Sclerosis

**DOI:** 10.3389/fimmu.2015.00565

**Published:** 2015-11-05

**Authors:** Kevin Blauth, Gregory P. Owens, Jeffrey L. Bennett

**Affiliations:** ^1^Department of Neurology, University of Colorado Denver, Aurora, CO, USA; ^2^Department of Ophthalmology, University of Colorado Denver, Aurora, CO, USA; ^3^Program in Neuroscience, University of Colorado Denver, Aurora, CO, USA

**Keywords:** multiple sclerosis, B cells, lymphocyte trafficking, chemokines, blood–brain barrier

## Abstract

B cells play a central role in multiple sclerosis (MS) pathology. B and plasma cells may contribute to disease activity through multiple mechanisms: antigen presentation, cytokine secretion, or antibody production. Molecular analyses of B cell populations in MS patients have revealed significant overlaps between peripheral lymphoid and clonally expanded central nervous system (CNS) B cell populations, indicating that B cell trafficking may play a critical role in driving MS exacerbations. In this review, we will assess our current knowledge of the mechanisms and pathways governing B cell migration into the CNS and examine evidence for and against a compartmentalized B cell response driving progressive MS pathology.

## Introduction

In recent decades, accumulating evidence has brought B cells into focus as critical players in multiple sclerosis (MS) pathogenesis. B cells are present at elevated levels in inflamed MS central nervous system (CNS) tissue and are significantly increased in MS cerebrospinal fluid (CSF) (1, 2). Furthermore, IgG is synthesized intrathecally in MS patients (3), and IgG and complement are characteristic features of both type 2 and active MS lesions (4–6). In the CSF, the presence of oligoclonal IgG bands (OCBs) are a long-standing hallmark of MS diagnosis, and in the meninges, B cell-predominant lymphoid aggregations [germinal center (GC)-like structures] are observed in some relapsing and secondary progressive patients (7, 8). Finally, clinical trials of the anti-CD20 monoclonal antibodies rituximab (9), and ocrelizumab (10), have demonstrated beneficial effects on MRI lesion load and relapse activity in MS patients.

Many questions about the role of B and plasma cells in MS remain unanswered. What factors drive B cells into the CNS, through which pathways do they travel, and are these cells persistent or transient? When during the course of disease do B cells populate the CNS and are there particular CNS niches in which B cells thrive? How may (GC)-like structures contribute to MS pathology? In this review, we will examine the chemotactic cues, migratory pathways, and CNS factors that facilitate B cells trafficking and survival in the inflamed CNS, and evaluate evidence supporting a compartmentalized B cell response in MS pathogenesis.

## B Cell Migration into the CNS in Health and Disease

### B Cells are Directed into the CNS by Chemokine Signaling

B cells may be observed in the healthy brain but are sparse in number, and increase drastically during neuroinflammation (11, 12). B cells express a robust array of chemokine receptors that largely dictate their movement, and the B cell chemokine receptor profile is dependent upon their state of differentiation and external microenvironment. The local milieu of cytokines in the inflamed CNS may also promote B cell migration by enhancing B cell chemoattraction and lymphoid organization. For instance, lymphotoxin-α expressed along the outer layer of inflamed vessel walls may facilitate lymphoid organogenesis and the formation of meningeal B cell GC-like structures (13).

Several chemokines and their receptors (in parentheses) have been shown to influence CNS B cell trafficking: CCL2 (CCR2, CCR3), CCL3 (CCR1, CCR5), CCL20 (CCR6), CXCL10 (CXCR3), CXCL12 (CXCR4, CXCR7), and CXCL13 (CXCR5) (Table 1). Among these factors, CXCL13 may play a central role. The CSF concentration of CXCL13 is elevated in MS patients (14), correlates with conversion from clinically isolated syndrome (CIS) to definite MS (15), and shows a strong correlation with B cell numbers in the CSF of MS and other neuroinflammatory diseases (14, 16). Indeed, nearly all CD19+ CSF B cells express the CXCL13 receptor, CXCR5 (14). Elevated CSF CXCL13 correlates strongly with the CNS accumulation of class-switched CD27+ memory B cells, CD27−IgD− B cells, and unswitched CD27+ memory B cells, but bears no relationship to the numbers of CD138 + CD38+ antibody-secreting plasmablasts and plasma cells (17). The ability of CXCL13 blockade to disrupt the formation of GC-like structures in the pancreatic islets of NOD mice suggests that meningeal B cell aggregates in MS patients may also develop from migrating memory B cells that differentiate intrathecally to plasmablasts and plasma cells (18).

**Table 1:** **B cell chemokines in multiple sclerosis**.

Chemokine	Levels in MS	Chemokine receptor	Reference
CCL2	Expressed by astrocytes and macrophages in acutely demyelinating lesions and active chronic lesions, at lesion edge, and in reactive astrocytes surrounding lesions	CCR2, CCR3	(19–22)
	Decreased in CSF	
CCL3	Unchanged in CSF	CCR1, CCR5	(19)
CCL20	Undetectable in CSF	CCR6	(23)
	Decreased in serum during relapse	
CXCL10	Increased in CSF	CXCR3	(19, 24)
	Upregulated in MS lesions	
CXCL12	Upregulated in chronic active and inactive MS lesions on astrocytes and blood vessels	CXCR4, CXCR7	(14)
CXCL13	Increased in actively demyelinating MS lesions, secreted by macrophages in the perivascular cuffs. Not present in chronic inactive lesions. Shown to be the most important determinant for B cell recruitment into the CNS.	CXCR5	(14, 16, 25)
	Increased in CSF during relapse and remission	
CX3CL1	Increased in CSF	CX3CR1	(26, 27)

As short-lived plasmablasts comprise a significant proportion of the CSF B cell population in MS (28), the chemokines CXCL10 and CXCL12 may also act as chemoattractants for CXCR3+ and CXCR4+ plasmablasts and additionally regulate the dynamics of CNS B cell trafficking in disease. Since CXCL10 is constitutively expressed by a subset of cells in the CNS subventricular zone, the gradient of CXCL10 may be a potent chemo-attractant signal for both activated T cells and antibody-secreting cells (29).

### Adhesion Molecules, B Cells, and the Blood–Brain Barrier

B cells follow chemokine gradients into the CNS via one of several anatomical pathways: (1) through the choroid plexus into the CSF; (2) through parenchymal vessels into the perivascular space; or (3) or through the post-capillary venules into the subarachnoid and Virchow–Robin spaces (30). B cells entering into the CNS through the choroid plexus must traverse apical tight junctions between epithelial cells composing the blood–CSF barrier, whereas B cells trafficking through parenchymal vessels or stromal venules ultimately need to traverse the tight junctions of the microvascular endothelial cells composing the blood–brain barrier (BBB). While the stages of lymphocyte transmigration across the blood–CSF barrier have yet to be described in detail, the sequence of leukocyte rolling, activation, arrest, crawling, and migration has been defined in great detail for blood–brain barrier trafficking (30). Basic adhesion molecule interactions important for T cell transmigration across the BBB include selectins during rolling (31), leukocyte very late antigen-4 (VLA-4) and endothelial vascular cell adhesion molecule-1 (VCAM-1) during the rolling and arrest, leukocyte lymphocyte function associated antigen-1 (LFA-1), and endothelial intercellular adhesion molecule-1 (ICAM-1) during arrest and migration (32, 33), as well as activated leukocyte cell adhesion molecule (ALCAM), and CD6 in migration (34).

The specific molecules required for B cell transmigration, however, are less clearly understood. Similar to requirements for T cell BBB transit, *ex vivo* studies using human adult brain-derived endothelial cells (HBECs) show that blockade of VLA-4, but not VCAM-1, inhibits B cell transmigration (35). Consistent with these findings, mice lacking the VLA-4 α-4 subunit specifically on B cells but not on other lymphocyte populations reduced disease severity significantly, and inhibited the recruitment of B cells into the CNS in an experimental autoimmune encephalitis model (36). In natalizumab-treated MS patients, CSF B and plasma cells are decreased in concert with the reduction in intrathecal CD4+ and CD8+ T cells (37). Complete (55%) or partial (27%) loss of CSF OCBs was observed in a natatlizumab-treated patient cohort following 2 years of therapy, suggesting that continuous trafficking of B cells to the CNS may be required to maintain the plasma cell niches producing intrathecal oligoclonal IgG (38). Antibody blockade of ICAM-1 and ALCAM also result in reduced migration of CD19+ B cells in *ex vivo* transmigration assays using HBECs as an artificial BBB (34, 35). The exact roles of ICAM-1 and ALCAM in CNS B cell trafficking *in vivo*, however, remain to be determined.

Recently, CNS meningeal lymphatic vessels containing T lymphocytes were discovered running parallel to the dural sinuses (39). These vessels drain to the deep cervical lymph nodes and may provide a novel route for trafficking B and T cells into or out of the CNS. This pathway may involve similar or distinct chemokine and adhesion molecules in the transit of various B cell populations that may infiltrate into the brain parenchyma, circulate in the CSF, populate GC-like structures, and transit back to the peripheral lymphoid compartment (39).

## Bidirectional B Cell Trafficking in MS

In general, lymphocytic surveillance of the healthy CNS is significantly lower than that of other peripheral organs (40). The majority of data, particularly in humans and mice, indicate that activated antigen-experienced T and B cells constitute almost the entirety (41) or the vast majority (17, 42) of the infiltrating lymphocytes. Whether activated lymphocytes return from the CNS compartment to the peripheral circulation has remained uncertain.

Recently, the ability of B cells to exit the CNS compartment and re-enter the peripheral circulation and, potentially germinal center responses, has been investigated by deep sequencing (43). Deep, or next-generation sequencing, allows for high-throughput recovery of B cell IgG heavy-chain variable region (VH) repertoires from patient fluids and tissues. When compared to single-cell methods, the large number of VH sequences analyzed by deep sequencing provides a more complete representation of the B cell Ig repertoire contained in a biological sample and substantially increase the likelihood of observing identical or related VH sequences between samples. This enhanced sensitivity likely accounts for the frequent identification of common peripheral and CNS B cell clones with deep sequencing (43–45) and the rare identification of those with single-cell analyses (46, 47).

**Figure 1:**
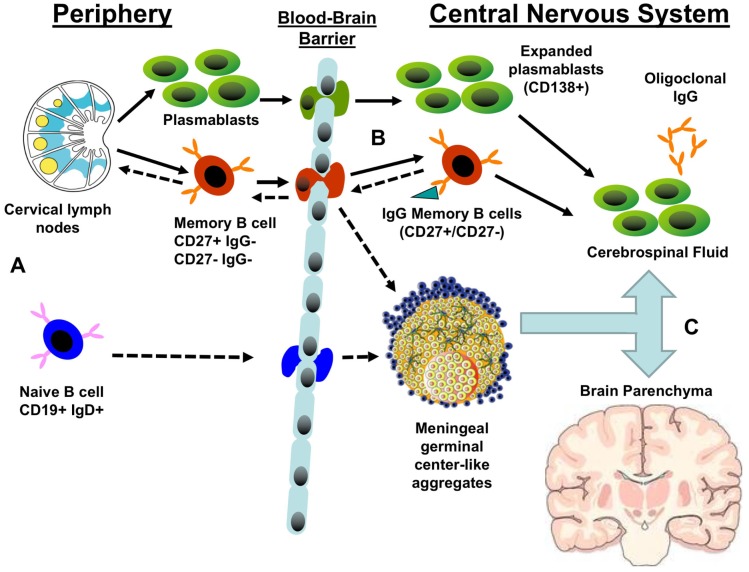
**Potential patterns of B cell trafficking in multiple sclerosis**. **(A)** The predominant stream of migratory B cells from the periphery to the CNS are likely to consist of either memory B cells or plasmablasts produced in the germinal centers of cervical lymph nodes. The presence of CSF B cell clones closely related to germline sequences suggests that naïve B cells may transit the blood–brain barrier to populate meningeal germinal center-like structures and produce CNS-restricted memory B cells. **(B)** Both migratory plasmablasts and memory B cells may contribute to the pool of central nervous system (CNS) antibody-secreting cells that produce the oligoclonal bands. Memory B cells may also enter germinal centers in meningeal lymphoid aggregates or draining cervical lymph nodes, resulting in further clonal expansion and affinity maturation. **(C)** A significant fraction of expanded B cell clones circulates between CNS compartments: cerebrospinal fluid, meningeal lymphoid aggregates, parenchymal lesions, and normal white matter. Solid arrows represent established pathways; dashed arrows represent putative pathways.

Using diverse strategies, patient populations, and methods, the VH repertoire from the peripheral blood, cervical lymph nodes, meninges, parenchyma, and CSF have been compared within the same MS patient (43–45). A common finding of each investigation was overlapping clonal B cell populations common to both the peripheral and CNS compartments. Overlapping peripheral blood and CSF B cell clones were observed among multiple subsets of Ig class-switched and post-germinal center B cells: CD27(+)IgD(−) memory B cells, CD27(hi)CD38(hi) plasma cells/plasmablasts, and CD27(−)IgD(−) negative memory B cells (44, 48, 49). While the number of overlapping sequences observed in each study varied due to technique and disease activity, lineage analysis of bi-compartmental B cell clones demonstrated patterns of somatic hypermutation consistent with bidirectional exchange (43–45). Some lineages showed a balanced distribution of peripheral and CNS compartment clones; while other lineages exhibited isolated CNS clones that were closely related to germline sequences. The pattern of overlapping B cell clones in these lineage trees suggest that B cells may travel back and forth across the BBB and re-enter germinal centers to undergo further somatic hypermutation (43–45) (Figure 1). In-depth analysis of the relationship between overlapping B cell clones in the cervical lymph nodes and CNS compartment of the same patient revealed that most of the shared VH clones were less mature sequences that originated, more often, in the periphery (45). More mature B cell clones tended to be restricted to either the peripheral lymph node or CNS compartment. Permutation testing supported a model in which B cell maturation into antibody-secreting cells occurs in both the periphery and CNS with antigen-specific maturation occurring in the periphery.

## Compartmentalization of the CNS B Cell Response in MS

A key question related to MS pathogenesis is whether B cell-mediated antigen-driven responses are generated, supported, and sustained within the CNS (43)? CNS B cells show evidence of clonal expansion (50, 51), and express somatically mutated, class-switched Ig transcripts (46, 52–55). As noted previously, B cells with clonally related VH sequences are recovered on both sides of the BBB; however, CNS B cells may eventually form a compartmentalized population that is independent of the peripheral B cell pool as disease progresses. Interestingly, compartmentalized CNS inflammation has been hypothesized to drive treatment-resistant progressive disease (56).

Oligoclonal CSF IgG (OCBs) are observed in over 95% of MS patients. The CSF Ig proteome and B cell Ig transcriptome show strong overlap, indicating that CSF B cell clones are a major contributor to MS intrathecal IgG (57). In a subsequent study, peptide sequences from the CSF Ig proteome were also found to match heavy- (VH) and light-chain (VL) transcriptome sequences recovered from the CNS parenchyma and CSF of the same individual (58). The CSF Ig proteome covered high percentages of VH (CNS-77%; CSF-84%) and VL (CNS-39%; CSF-60%) transcriptome sequences in one patient and were somewhat limited in a second due to low CSF Ig quotient (58). The results indicate that B cells and IgG in MS CSF accurately mirror the humoral immunity present at the site of brain tissue damage (Figure 1). Indeed, 39–62% of the B cell transcriptome sequences recovered from the meninges, demyelinating plaques, normal appearing white matter, and CSF of the same MS patient were shared between intrathecal compartments, indicating that a significant fraction of intrathecal B cells trafficked through the CNS (59). Some expanded B cells clones, however, appear restricted to regions of MS plaque and meninges, suggesting some potential for localized tissue injury (59).

Interestingly, recent studies have questioned whether the CSF-restricted OCBs identified by isoelectric focusing are truly exclusive to the CNS (60). While the majority of CSF OCBs matched IgG-VH transcripts only recovered from the CSF B cell transcriptome, several OCB peptides matched bi-compartmental peripheral blood and CSF VH sequences. Although the type of MS and disease therapies were not reported, lineage tree analysis of bi-compartmental B cell populations suggested that these B cell groups underwent immune stimulation on both sides of the BBB (Figure 1). As a result, there remains the possibility that CNS immune populations may maintain molecular links with the periphery despite contrary data from isoelectric focusing.

Ig VH gene usage from the periphery and CNS provides additional data supporting compartmentalization of the humoral immune response in MS patients. The analysis of Ig VH sequences from demyelinating plaques and CSF of affected individuals reveal substantial VH4 family bias compared to normal VH4 prevalence (61, 62). Similar to patients with viral meningitis, CNS VH4 germline sequences displayed evidence of clonal expansion and extensive somatic mutations consistent with antigen selection (53, 54). MS patient with the longest disease course had the largest number of distinct IgG clonal populations, while the patients with recent diagnoses had limited clonal populations. CSF B cells from patients with a single demyelinating event (clinically isolated syndrome) also showed clonally expanded, somatically hypermutated VH genes (63, 64). Interestingly, both the overrepresentation of VH4 family sequences (65) and a unique pattern of somatic hypermutation “antibody gene signature” (66) within the CSF Ig VH transcriptome predicted transition to clinically definite MS. Recent deep sequencing of MS CSF VH repertoires from six MS patients has also revealed an overrepresentation of VH4-39, VH4-59, and VH4-61 heavy-chain sequences. The bias of MS CSF B cell heavy chains to VH4 germline sequences suggests that their basic structure may define an antigen-binding pocket that favors interaction with target antigen(s). As a result, a compartmental CNS humoral immune response may be able to drive CNS injury independent of peripheral immune activity.

Lastly, the GC-like structures or lymphoid infiltration have been noted in a large proportion of meningeal tissue from secondary progressive early stage cortical biopsies (7, 8, 67, 68). These CNS-specific immune infiltrates correlate with the severity of disease progression (8) and are associated with cortical neuronal loss in adjacent gray matter (69). The composition of these infiltrates included B cells, T cells, and dendritic cells, whose organization may resemble lymphoid follicles (7, 67). In addition, the presence of IgG and CXCL13 (7, 67) provide additional information, suggesting the active attraction and maintenance of B cells in MS meninges. The identification of CD19 + CD38hiCD77 + Ki67 + Bcl2− centroblasts in the CSF but not the peripheral blood of MS patients suggests that a compartmental humoral immune response in the MS CNS recapitulates all stages of B cell differentiation and may create a self-sufficient CNS response that is independent of the immune activity in the periphery (13). Additional data, however, are required to establish the relationship between the generation and maintenance of meningeal GC-like structures, intrathecal B cell clonal populations, and progressive disease (Figure 1). Peripheral B cell depletion, effective in early phase clinical trials in relapsing MS (9, 10), has not delivered similar efficacy for the treatment of primary progressive disease (70). This could be directly related to the inefficient depletion of the intrathecal B cell population in progressive (71) versus relapsing MS (72) due to compartmentalization of the B cell response in progressive disease and inefficient transit of anti-CD20 monoclonal antibody across the BBB. Interestingly, intrathecal administration of anti-CD20 monoclonal antibody rapidly depleted both peripheral and CD19+ B cells within days of delivery (73). Therefore, intrathecal anti-CD20 therapy may offer a novel avenue to evaluate the role of intrathecal B cell inflammation in progressive disease. The recent development of novel MRI techniques to identify meningeal follicles may offer a non-invasive tool to correlate therapeutic response with changes in meningeal inflammation (74).

## Conclusion

Molecular analysis of the B cell response in MS has demonstrated that antigen-experienced B cells are shared between multiple CNS compartments and the peripheral immune response. Several features of CNS clonal B cell populations suggest that B cell subsets may not be shared between the CNS and periphery as disease progresses and that meningeal GC-like structures may support an independent, compartmentalized immune response that is correlative with measures of CNS injury. The data supporting the trafficking of B cells back and forth across the BBB are undermined by the technical constraints of single-cell PCR, deep sequencing, and sampling errors. For instance, the VH sequences defining the bi-compartmental B cell clones may be skewed by errors in PCR sequencing, multiple cDNA copies from the same cell, errors in flow cytometry, or limited blood and CSF sampling. Future studies are needed to confirm present data using defined MS cohorts at multiple stages of disease. The influence of current MS therapeutics on B cell trafficking and survival may be critical for understanding MS pathogenesis and establishing biomarkers of disease activity and therapeutic efficacy.

## Author Contributions

Drs. KB, GO, and JB participated in the analysis, interpretation, writing, and critical review of the manuscript for important intellectual content.

## Conflict of Interest Statement

The authors declare that the research was conducted in the absence of any commercial or financial relationships that could be construed as a potential conflict of interest.
